# Comparison of efficacy between brachytherapy and penectomy in patients with penile cancer: a meta-analysis

**DOI:** 10.18632/oncotarget.18761

**Published:** 2017-06-28

**Authors:** Xiheng Hu, Jianghai Huang, Sailan Wen, Jun Fu, Minfeng Chen

**Affiliations:** ^1^ Department of Urology, Xiangya Hsopital, Central South University, Changsha, Hunan Province 410008, China; ^2^ Department of Pathology, The Second Xiangya Hospital of Central South University, Changsha, Hunan Province 410011, China; ^3^ Department of Oncology, Xiangya Hospital, Central South University, Changsha, Hunan Province 410008, China

**Keywords:** brachytherapy, penectomy, penile cancer, meta-analysis

## Abstract

We conducted a meta-analysis to compare the efficacy of brachytherapy and penectomy in patients with penile cancer. We searched the published articles in the PubMed, Web of Science, China National Knowledge Infrastructure, and Wanfang databases up to March 20, 2017. Twenty-two studies entered the final analyses. We used five-year overall survival rate, five-year local control rate, disease-free progression and lymph node positive rate to assess the efficacy. The meta-analysis found that patients who received penectomy had higher five-year local control rate (85% vs 80%, odds ratio = 0.72, 95% confidence interval: 0.58–0.90), five-year disease-free progression rate (77% vs 72%, odds ratio = 0.77, 95% confidence interval: 0.63–0.93) and lymph node positive rates (24% vs 20%, odds ratio = 0.79, 95% confidence interval: 0.64–0.98) than brachytherapy. No significant difference was observed for two group in five-year overall survival rate (76% vs 74%, odds ratios = 1.11 with the 95% confidence interval: 0.91–1.36). Both of penectomy and brachytherapy can improve the survival status. Penectomy provided better control efficacy, and not improved the survival status compared with brachytherapy solely. However, further research was required because of retrospective nature and potential bias of the data.

## INTRODUCTION

Penile cancer was a relatively rare cancer, and its morbidity rate accounted 0.4%–0.6% of all tumors. Comparing to developed countries, penile cancer’s incidence was significantly higher in developing countries [1]. The main risk factors of penile cancer mainly attribute to redundant prepuce, excessive sexual partners, HPV infection, and so on [2]. As a cancer of male sex organs, penile cancer not only threatens males’ physical health and life, but also severely affect their psychological health, social functions and life quality [3]. Currently, the major treatment for penile cancer was surgical operation, and along with the development of laparoscopic technique, the indications and methods of laparoscopic inguinal lymph node dissection had been considered and beat debated among doctors [4, 5]. Besides the surgical operation approach, radiotherapy and chemotherapy were gradually applied in clinical work.

The extent of nodal involvement is the strongest influencing factor for disease-specific mortality in penile cancer. Tumor grade and number of involved inguinal nodes are important predictors of pelvic lymph node involvement [6]. The recommended treatment plan was full or partial penectomy. Though penectomy was effective for lesions control, this surgery broken penis morphology, usually accompanied by some mental illnesses and social dysfunction such as depression, suicide [7]. Penectomy may not be a preference choice under some conditions. Brachytherapy, as a treatment way of retaining the full organ, had been available for several decades. It was reported that the five-year survival rate was 87%, and preservation rate was 88% in patients with T1–T2 stage who received brachytherapy solely. Crook found that brachytherapy could treat early stage penile cancer and achieve similar benefits with penectomy [8]. As opposed to this result, Sarin reported efficacy of radiation was worse than those with penectomy [9]. Some other studies also gave different results based on different sample size, power and patients’ selection. Data from randomized controlled trials, prospective studies were few. In the present study, we undertook a meta-analysis to assess the overall survival rate, local control rate, and disease-free survival rate of brachytherapy and penectomy in patients with penile cancer.

## RESULTS

### Study selection

**Figure 1:**
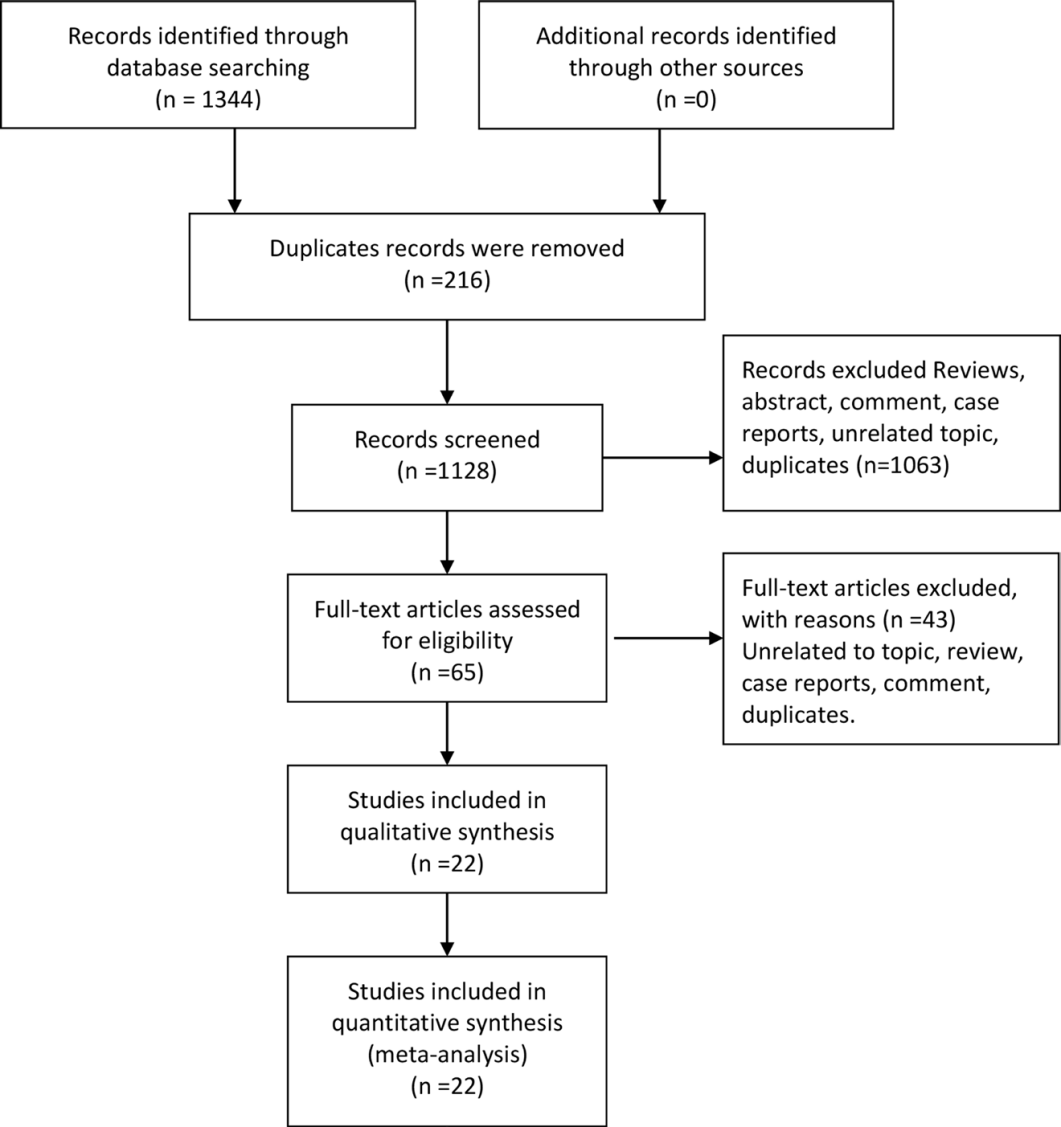
Flow chart of study selection

The flow of study selection was presented in Figure 1. Our initial search returned 1344 records. We got 1128 records after duplicates records were removed. 1063 records were excluded after scanning the abstracts and titles, and 65 articles were potentially eligible for inclusion. Then we read the full-text articles assessed for eligibility, and 22 articles were finally included for qualitative and quantitative synthesis [8–28].

### General characteristics and assessment of quality

The general characteristics of the include studies were summarized in Table 1. These articles were published from 1992 to 2016. The sample size ranged from 23 to 642, with the number of 2560 patients. Among the included studies, eleven of these studies solely were about brachytherapy, two studies compared brachytherapy and penectomy [8, 9], and nine solely were about penectomy. For the purposes of meta-analysis, five-overall survival rate, five-local control rate, disease-progression rate, and lymph node positive rate were combined and compared between brachytherapy and penectomy. According to the assessment scale, the mean score of included studies was 7.1, located in a high quality. The Supplementary Table 1 gives the specific description of scale.

**Table 1:** General characteristics of included studies in the meta-analysis

Author	Year	5-year OS (%)	5-year LC (%)	Disease-free progression (%)	Lymph node positive	Sample size
Crook et al.	2009	81	88	70	0.08	60
Soria et al.	1997	63	77	54	0.23	72
Delaunay et al.	2013	81	84	-	0.00	47
Rozan et al.	1995	68	78	59	0.19	174
Chaudhary et al.	1999	72	70	78	0.35	23
Garcia et al.	2012	82	76	71	0.00	21
Mazeron et al.	1984	79	78	76	0.10	50
Delannes et al.	1992	85	82	83	0.24	51
Kiltie et al.	2000	69	81	76	0.00	31
De Crevoisier et al.	2009	-	80	86	0.18	144
Cordoba et al.	2016	82	74	64	0.38	73
Guimaraes et al.	2009	84	83	76	0.24	333
Lont et al.	2006	-	88	88	0.17	100
Ozsahin et al.	2006	53	88	87	0.30	23
Zouhair et al.	2001	61	75	75	0.29	29
Mistry et al.	2007	83	88	87	0.23	24
Kattan et al.	2006	58	-	-	0.20	175
Phillippou et al.	2008	89	86	75	0.28	179
Omellas et al.	2008	75	-	75	0.44	642
Du et al.	2003	38	-	38	0.13	76
Lei et al.	2016	78	-	85	0.16	129
Kong et al.	2002	80	-	78	0.24	104

5-year OS, 5-year overall survival; 5-year LC, 5-year local control.

### Pooled outcomes

**Figure 2:**
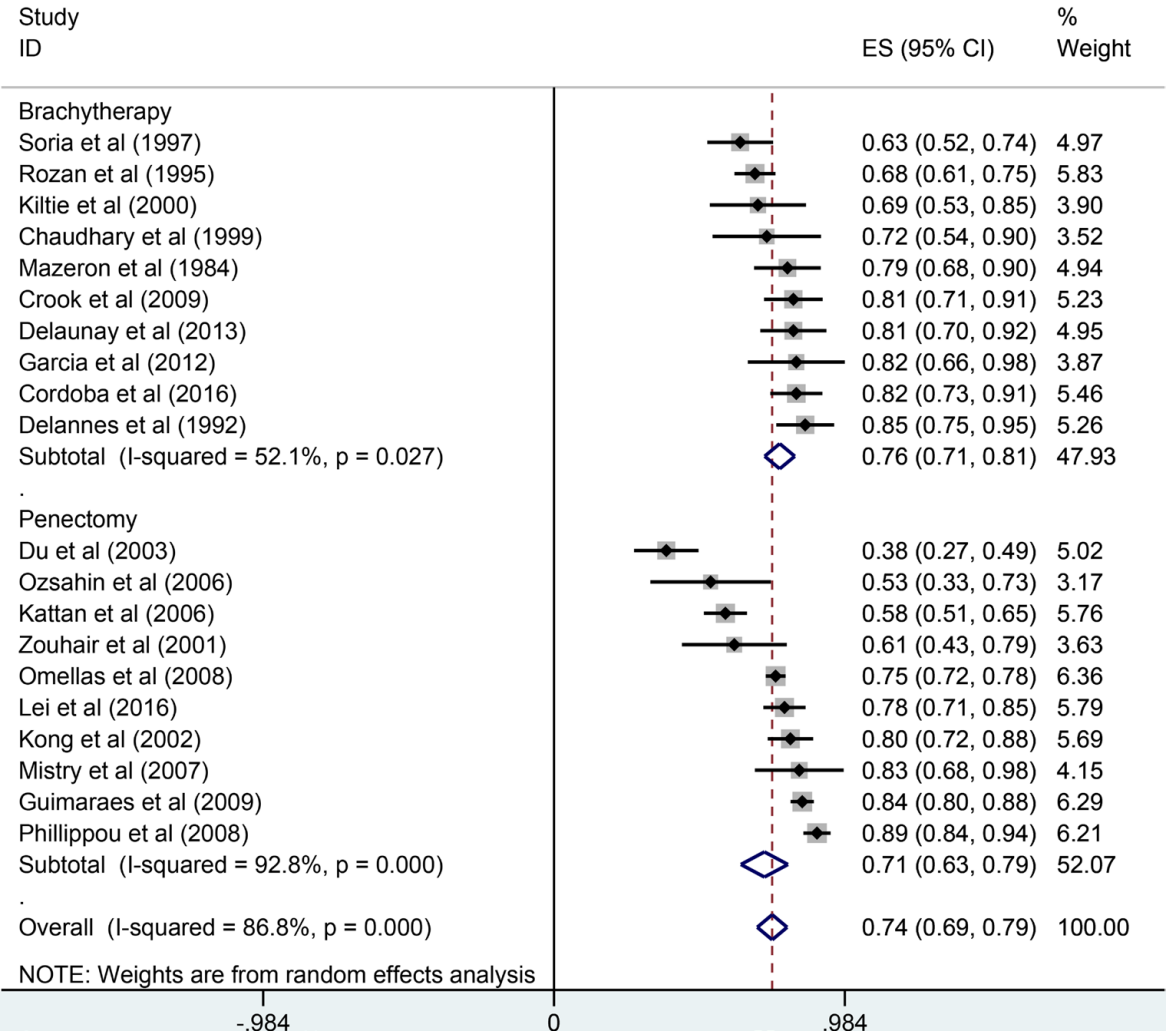
Comparisons of 5-year overall survival between brachytherapy and penectomy

**Figure 3:**
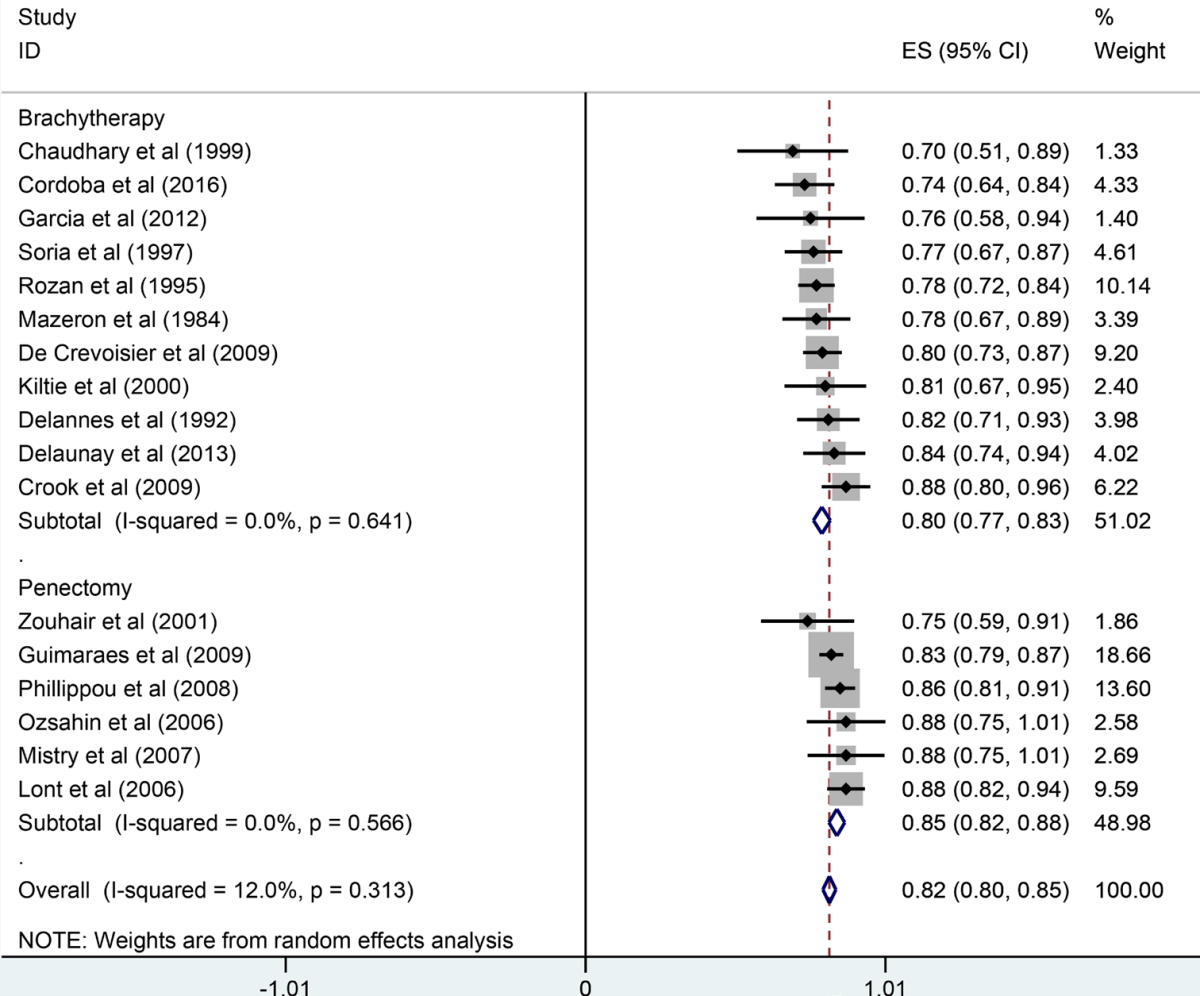
Comparisons of 5-year local control between brachytherapy and penectomy

**Figure 4:**
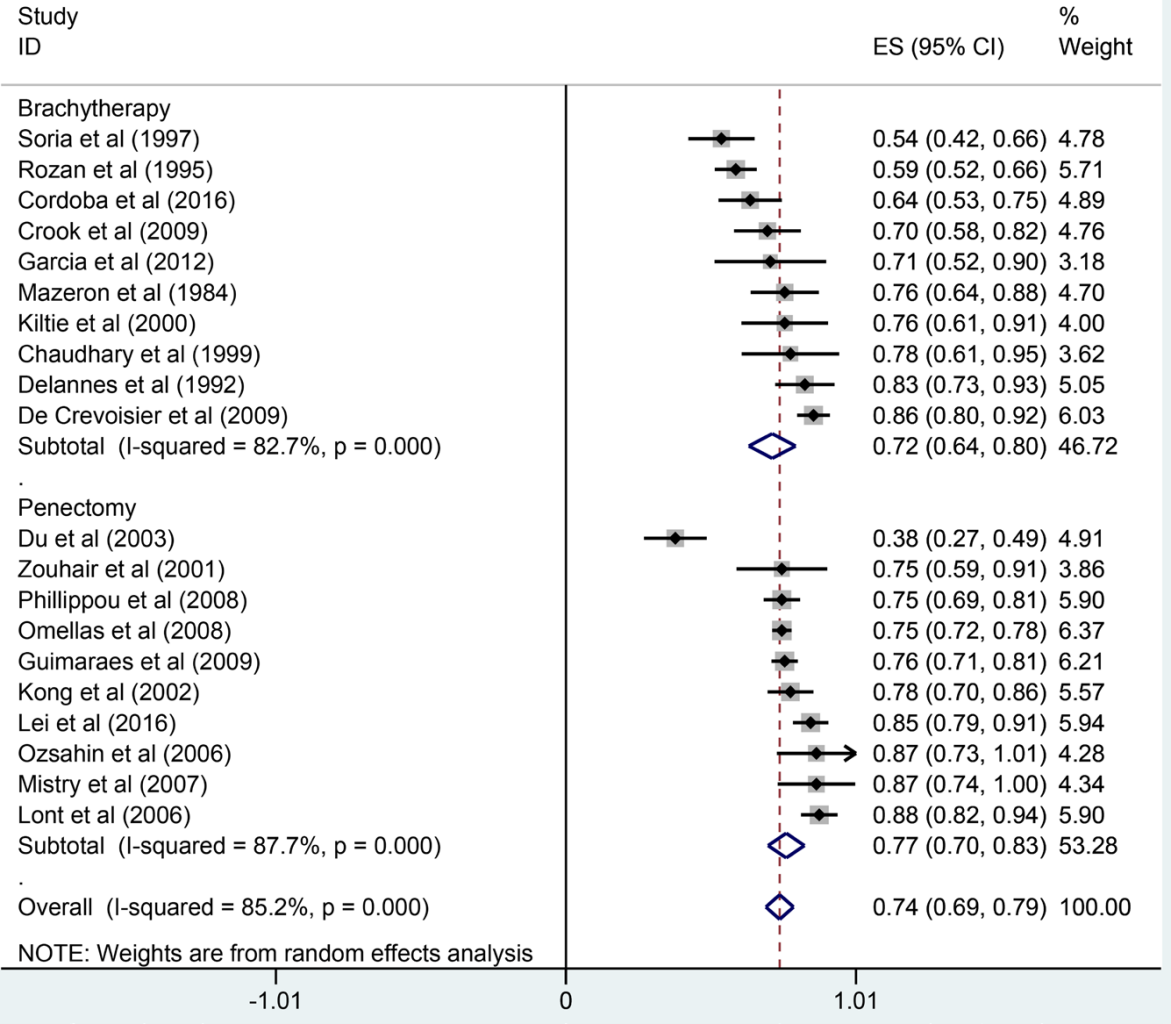
Comparisons of disease-free survival between brachytherapy and penectomy

**Figure 5:**
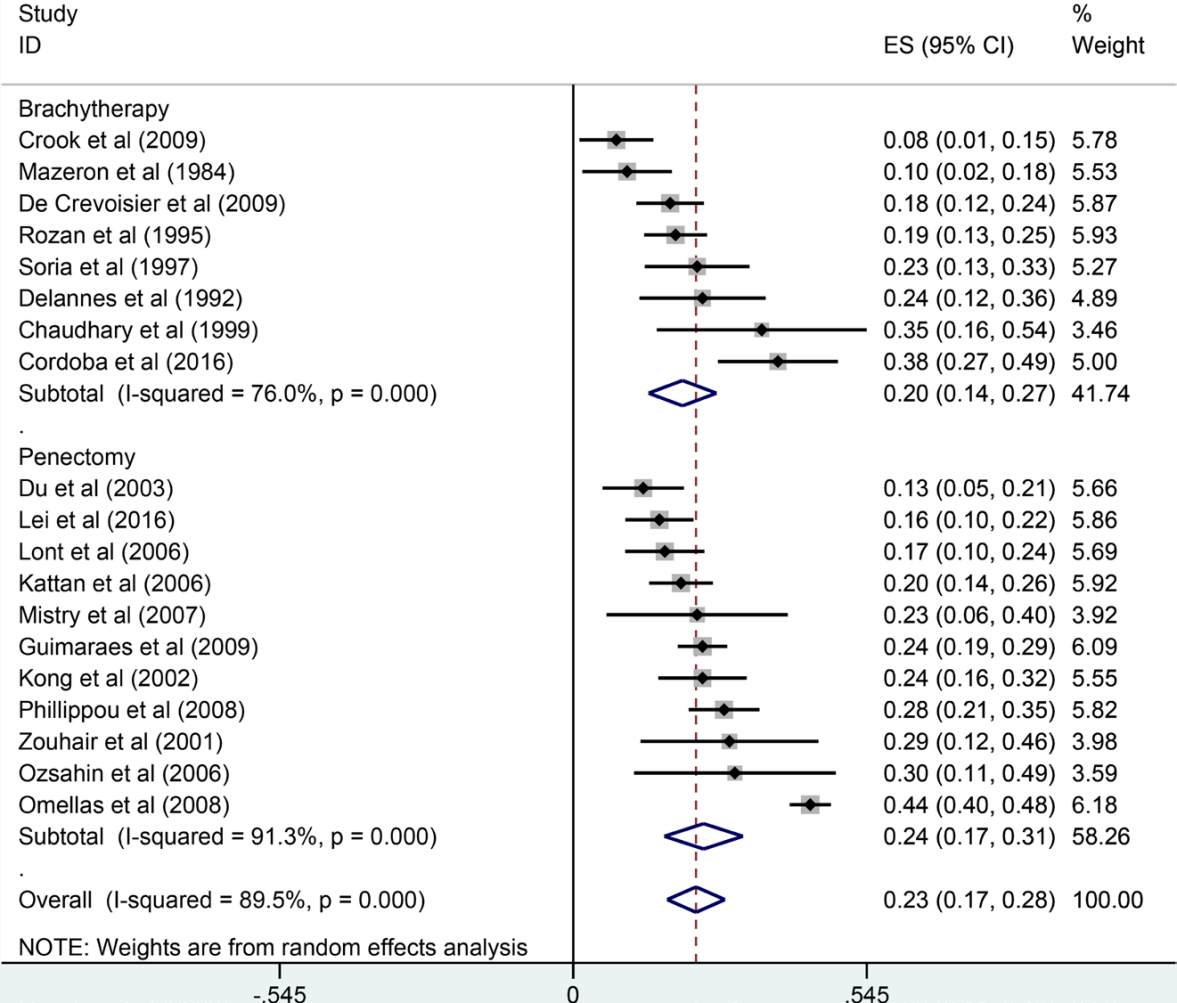
Comparisons of lymph node positive between brachytherapy and penectomy

The pooled results were presented in Table 2. Twenty studies reported five-year overall survival rates. The Figure 1 presented the five-year overall survival rates of brachytherapy and penectomy (76% vs 74%, Figure 2). The random-effect models result found that there was no statistically significance in the five-year overall survival rate, the combined odds ratios (OR) was 1.11 with the 95% confidence interval (CI) of 0.91–1.36 (*P* > 0.05). We also calculated the five-year local rates. Eleven studies in brachytherapy group and six studies in penectomy group reported that five-year local control rated in brachytherapy group. The five-year local control rates of brachytherapy group were obviously lower than that of penectomy group (85% vs 80%, OR = 0.72, 95% CI: 0.58–0.90, *P* = 0.003, Figure 3). Ten studies in brachytherapy group and eleven ones in penectomy groups reported that for disease-free survival rates, the penectomy group was still higher than that of brachytherapy (77% vs 72%). The significant difference was observed (OR = 0.77, 95% CI: 0.63–0.93, Figure 4). Parallel with disease-free survival, the lymph node positive rates of penectomy was higher than that of brachytherapy (24% vs 20%, OR = 0.79, 95% CI: 0.64–0.98, *P* = 0.028, Figure 5). The power of meta-analysis ranged from to 78 to 89%.

**Table 2:** Comparisons of outcomes between brachytherapy and penectomy

Outcomes	Brachytherapy	Penectomy	Odds ratio	*P*
Overall	746	1814		
5-year overall survival	0.76 (0.71–0.81)	0.74 (0.69–0.79)	1.11 (0.91–1.36)	0.284
5-year local control	0.80 (0.77–0.83)	0.85 (0.82–0.88)	0.72 (0.58–0.90)	0.003
Disease-free survival	0.72 (0.64–0.80)	0.77 (0.70–0.83)	0.77 (0.63–0.93)	0.008
Lymph node positive	0.20 (0.14–0.27)	0.24 (0.17–0.31)	0.79 (0.64–0.98)	0.028

We conducted sensitivity analyses via excluding one study each time. The results did not alter (data did not show.) We used the Begg’s and Egger’s test to evaluate the publication bias. The results did not indicate the existence of publication bias (*P* = 0.256, *P* = 0.141). The Supplementary Figure 1 give details. Dots scattered around the straight line, and there was no obvious publication bias.

## DISCUSSION

Our meta-analysis found that (1) penectomy and brachytherapy could improve five-year overall survival rate in penile cancer patients, and no obvious difference was observed between two treatment ways. (2) Penectomy compared with brachytherapy significantly increased local control rate for patients with penile cancer; (3) penectomy further improved five-year disease-free progression rate.

Five-year overall survival rate was almost equal for both penectomy and brachytherapy. But these two treatment ways were significantly higher than reported five-year overall survival rate via external-beam radiotherapy. Ozsahin reported that the five-year overall survival rate of patients with radiotherapy was 56% [29]. Other similar findings ranged from 55 to 75% [30, 31]. According to previous reports, patients with nodal disease tend to choose surgery treatment. This higher proportion of lymph nodal positives may mask otherwise superior survival status. However, patients who received brachytherapy had higher risks of tumor recur, they finally received surgery treatment. That may explain why equivalent survival rate was high although penectomy group had a relative high lymph nodal positive rate. Sharma reported that three-year survival rate of patients with brachytherapy was 83%, 93% of patients kept pennies, and only two cases recur. This study indicated that brachytherapy was efficient for penile cancer with stage 1–2, toxicity of treatment was acceptable, and life of patients was improved [32]. Considering the relative present of noninvasive and low-grade tumors, brachytherapy treatment may be a prior option for patients with node negative, T1/T2 or grade 1 disease. This treatment way reduced complications such as social dysfunction, psychological problems (depression, suicide). As opposed to this, surgical treatment could be acceptable for lymph node positive, T3/T4 disease types.

Compared with partial resection, penectomy could clear focus well. This treatment usually leads to loss of sexual function, and affected social activity and life quality. Reservation form and function of penile become important considerations. Rosa reported that recur rate of patients with partial resection in 859 patients was 27%, the partial group was significantly higher than that of penectomy [33]. However, there were no significantly difference in five-year survival rate between two groups. Tumor grade [34], lymph nodes positive, and regional lymph node metastasis were associated with survival status [35]. Therefore, keeping the penile was required for patients’ life quality. Complications caused by radiotherapy were penile parenchyma necrosis, and the incidence ranged from 0 to 21%. This range fluctuated widely. These were probably associated with process and doses of radiotherapy [36]. It was reported that brachytherapy caused higher risks of necrosis than external beam radiation, especially more than 60 Gy or tumor with T3 stage. Adel reported such a case with penile necrosis after radiation therapy. Pathology results suggested that the cancer was squamous cell carcinoma with T2. The patients still did not make it after penectomy because of sepsis lead by extensive gangrene [37]. This case implies that full evaluation for complications and efficacy was essential. Brachytherapy was still a prior option for early penile cancer because of preserving function and form.

One of the main strength of the current meta-analysis was compliance with the Preferred Reporting Items for Systematic Reviews and Meta-Analysis Protocols and some guidelines recommended by the Cochrane collaboration. The other was combined limitations of this study also need to be addressed. The study included in the meta-analysis were performed in different patients’ settings. Studies from penectomy group included both patients with full or partial penectomy, and results were not presented separately between two groups. In parallel with this point, the brachytherapy group consisted of some patients with adjuvant radiation or lymph node dissection. Different population settings could underestimate or overestimate the results. Meanwhile, some potential selection bias could exist, which would likely never be required. There were some discrepancies within included studies in the meta-analysis such as sample size, patient’s selection. Besides, different tumor grade within studies could lead to some bias. The brachytherapy group usually were in stage one, and penectomy group tend to be the relative advanced stage. We also found the lymph node positive rated in penectomy group was higher than that in the brachytherapy (20% vs 24%). We did conduct subgroup analyses of exploring the heterogeneity because relevant information was unavailable.

In conclusion, both of penectomy and brachytherapy can improve the survival status. But there was no significantly difference in overall survival rate between penectomy and brachytherapy. Penectomy provided better control efficacy. However, further research was required because of retrospective nature and potential bias of the data. In the future clinical practice, preoperative and postoperative adjuvant radiotherapy should be taken into consideration. Patients could benefit a lot from combination therapy.

## MATERIALS AND METHODS

No ethical approval was involved for this second study based on the published studies. We undertook this meta-analysis in accordance with the Preferred Reporting Items for Supplementary Table 2 Systematic Reviews and Meta-Analysis Protocols (PRISMA-2009) [38].

We systematically searched the published articles in the PubMed, Web of Science, China National Knowledge Infrastructure, and Wan Fang databases up to March 20, 2017. The following keywords were used: penile cancer, penis squamous cell carcinoma, brachytherapy, radiotherapy, penectomy, and surgery. We placed some restrictions on the language in English and Chinese but date of publication.

### Criteria for study selection

The selected studies must meet the following criteria: study design based on randomized clinical trials or cohort study; patients with I–III stage received brachytherapy or penectomy or comparing brachytherapy and penectomy. Followed up for at least one year; provided one of the following at least one of outcomes: five-year overall survival, five-year local control rate, disease-free progression, lymph node reporting; If two or more studies reported the same data, we selected the study with the larger sample size.

### Data extraction

Two investigators independently extracted the required information using a standardized excel sheet. We collected the following information from each study: year of publication, first author, sample size, five-year overall survival, five-year local control, disease-free progression, lymph node positive or not. We tried our best to contact the authors for requiring relevant information if necessary.

We used the Newcastle-Ottawa Scale to evaluate the quality of included study [39]. This scale included three main items, and seven sub-items: selection (exposed cohort, non-exposed cohort, ascertainment of exposure, outcome of interest), comparability, outcomes (assessment of outcome, length of follow-up, adequacy of follow-up). We assigned quality categories based on the scores of each study. We separated three levels: high quality (7–9 scores), medium quality (4–6 scores) and low quality (less than 4 scores). We resolved discrepancies by consensus.

### Statistical analysis

We firstly estimated the five-year overall rate, five-year local control rate, disease-free progression rate by using inverse arcsine variance weights for random-effect models. We compared the five-year overall rate, five-year local control rate, disease-free progression risk of brachytherapy and penectomy. The relevant odds ratios with 95% confidence intervals were calculated. We used the random-effect model proposed by DerSimonian and Laird to compare the efficiency between the brachytherapy and penectomy [40]. We used Cochrane Chi-square test and I^2^ statistic to assess the heterogeneity within studies. We defined high, medium, and low heterogeneity as 75%, 50%, and 25%, respectively [41]. If *P* value was less than 0.1, we assumed there was heterogeneity within study. Because the number of studies included in the meta-analysis was less than ten. It was inappropriate to assess the publication bias using funnel plot. Therefore, we used Begg’s and Egger’s test to evaluate the publication bias [42, 43]. All statistical analyses were performed on STATA 12 version platform. *P* < 0.05 was considered as significance.

## SUPPLEMENTARY MATERIALS FIGURE AND TABLES





## Abbreviations

OR: odds ratio
CI: confidence interval
PRISMA-2009: Preferred Reporting Items for Systematic Reviews and Meta-Analysis Protocol
