# Supplementary material for: Comparison of efficacy between brachytherapy and penectomy in patients with penile cancer: a meta-analysis
Source: Oncotarget. 2017 Jun 28;8(59):100469–77. doi: 10.18632/oncotarget.18761 (PMC5725035; doi:10.18632/oncotarget.18761)
Supplement: Supplementary file 1 [file oncotarget-08-100469-s001.pdf]

Comparison of efficacy between brachytherapy and penectomy in patients with penile cancer: a meta-analysis

Supplementary Materials

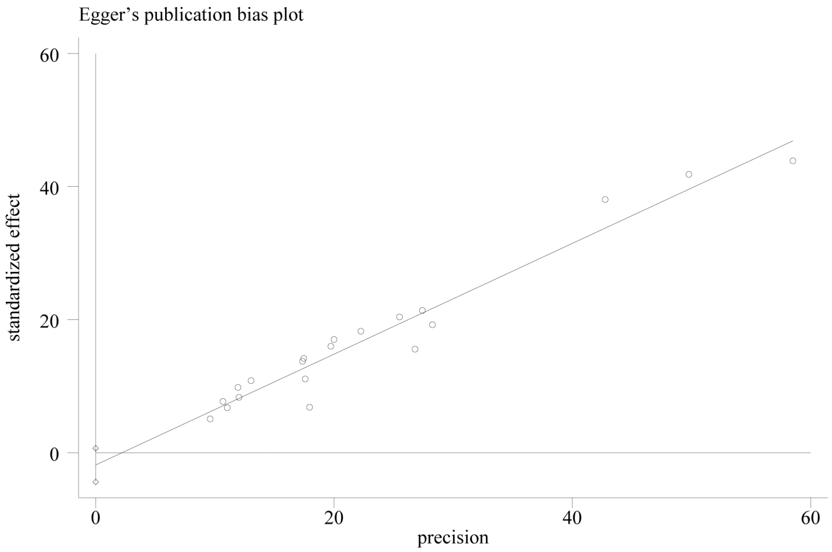

Supplementary Figure 1: Detection of Publication bias (Dot Scattered around in the straight line when there was no publication bias).

Supplementary Table 1: Methodological quality assessment (risk of bias) of included studies by Newcastle-Ottawa Scales

Study	Selection				Comparability	Outcome			Total score
	Exposed Cohort	Nonexposed Cohort	Ascertainment of exposure	Outcome of interest		Assessment of outcome	Length of follow-up	Adequacy of follow-up	
Du et al.	*	-	*	*	**		*	*	6
Lei et al.	*	-	*	*	*	*	*	*	6
Lont et al.	*	-	*	*	**	*	*	*	7
Kattan et al.	*	-	*	*	**	*	*	*	7
Mistry et al.	*	-	*	*	**	*	*	*	8
Guimaraes et al.	*	-	*	*	*	*	*	*	6
Kong et al.	*	-	*	*	**		*	*	6
Phillippou et al.	*	-	*	*	*	*	*	*	7
Zouhair et al.	*	-	*	*	**	*	*	*	8
Ozsahin et al.	*	-	*	*	*	*	*	*	7
Omellas et al.	*	*	*	*	**	*	*	*	8
Garcia et al.	*	-	*	*	*	*	*	*	7
Kiltie et al.	*	-	*	*	*	*	*	*	6
Delaunay et al.	*	-	*	*	*	*	*	*	7
Crook et al.	*	-	*	*	**	*	*	*	8
Mazeron et al.	*	*	*	*	*	*	*	*	7
De Crevoisier et al.	*	-	*	*	*	*	*	*	8
Rozan et al.	*	-	*	*	*	*	*	*	7
Soria et al.	*	-	*	*	**	*	*	*	8
Delannes et al.	*	-	*	*	**	*	*	*	8
Chaudhary et al.	*	-	*	*	**	*	*	*	8
Cordoba et al.	*	-	*	*	*	*	*	*	7

Supplementary Table 2: PRISMA checklist. See Supplementary_Table_2.
